# Lacto-Peptine

**Published:** 1874-11

**Authors:** Laurence Alexander

**Affiliations:** Yorkville, S. C.


### LACTO-PEPTINE.

By LAURENCE ALEXANDER, M.D., of Yorkville, S. C.

*Editor Atlanta Medical and Surgical Journal:*

Some time ago a small box, labelled "Physicians' Sample Lacto-peptine," was placed in my hands, with the request that I would give it a trial upon some one suffering from dyspepsia. Having, like other physicians, a large *per centum* of just such cases always on hand, in which various medicines and remedies had been used without success, I gladly consented, hoping that something had really been found at last to supply the want felt by every practitioner in the treatment of this troublesome complaint. After several months' experience in the use of this preparation, in which it has been thoroughly tested upon a large number of patients with such gratifying results, I am induced to recommend it to the consideration of the profession, feeling confident that, with due care in their diagnosis, and the many little cautions always necessary, such as restricting the excessive use of fluids while eating, etc., and a little patience on the part of the sufferer, its good effects will be seen beyond a doubt.

While I employ it extensively in many deranged conditions of the bowels incident to infancy and childhood, I find it equally efficacious in constipation and all diseases arising from imperfect nutrition in the adult. In sickness of pregnancy it answers well, far exceeding, in my hands, oxalate of cerium, extract lupulin, or the drop doses of carbolic acid, so highly extolled by some practitioners. In its combination with iron, quinine and strychnia, we have the advantage of using, in cases of great nervous depression and debility peculiar to the dyspeptic, our most valuable agent in a truly elegant form.

